# Insights into Chromatin Structure and Dynamics in Plants

**DOI:** 10.3390/biology2041378

**Published:** 2013-11-28

**Authors:** Stefanie Rosa, Peter Shaw

**Affiliations:** Department of Cell and Developmental Biology, John Innes Centre, Norwich Research Park, Colney, Norwich NR4 7UH, UK; E-Mail: stefanie.rosa@jic.ac.uk

**Keywords:** chromatin, histone modifications, histone variants, gene positioning, nuclear structure, chromosome territories, gene expression

## Abstract

The packaging of chromatin into the nucleus of a eukaryotic cell requires an extraordinary degree of compaction and physical organization. In recent years, it has been shown that this organization is dynamically orchestrated to regulate responses to exogenous stimuli as well as to guide complex cell-type-specific developmental programs. Gene expression is regulated by the compartmentalization of functional domains within the nucleus, by distinct nucleosome compositions accomplished via differential modifications on the histone tails and through the replacement of core histones by histone variants. In this review, we focus on these aspects of chromatin organization and discuss novel approaches such as live cell imaging and photobleaching as important tools likely to give significant insights into our understanding of the very dynamic nature of chromatin and chromatin regulatory processes. We highlight the contribution plant studies have made in this area showing the potential advantages of plants as models in understanding this fundamental aspect of biology.

## 1. Introduction: Chromatin and Nuclear Architecture

In eukaryotic cells the nuclear DNA does not appear naked but is associated with many proteins to form the complex called chromatin. The most prominent proteins in chromatin are the histone proteins—basic proteins responsible for the vast degree of packaging of the DNA within the confines of the eukaryotic nucleus. Approximately 146 base pairs of DNA are wrapped around an octamer of histones containing two copies of each of the four core histones (H2A, H2B, H3 and H4) forming the most fundamental unit of chromatin—the nucleosome [1]. 

Core histones have been structurally conserved through evolution and have evolved to accomplish two conflicting and yet vital tasks: on one hand the long DNA molecules have to be packaged within the limits of the eukaryotic nucleus, preventing knots and tangles and protecting the genome from physical damage; on the other hand the information that is encoded in the DNA needs to be accessed at appropriate times. These functions are, at least in part, regulated by local changes in nucleosome and chromatin structure by complex mechanisms that have only recently begun to be understood*.*

The nucleosomes are positioned every ~200 bp on a typical DNA strand, and can be linked together by the linker histone H1. Electron microscopy of isolated polynucleosomes clearly reveals the open “beads-on-a-string” structure of the so-called 10 nm DNA fibre [2] as first presented in electron micrographs by Don and Ada Olins and Chris Woodcock in the meeting for American Society of Cell Biology in 1973 [3,4]. It was also proposed that an early stage in compaction is the formation of a fibre with approximately 30 nm in diameter, in what can be designated a secondary structure of chromatin [5]. Several models have been suggested to describe the general structure of the 30 nm fibre: the one-start solenoid [5,6,7]; the two-start helix zigzag [8,9]; the cross-linker [10], and the supranucleosome [11]. Even though several models have been proposed, the arrangement of nucleosomes within the fibre is unknown and the occurrence of a 30 nm fibre has been seriously questioned [12]. Based on new approaches such as chromatin conformation capture and cryo-electron microscopy some authors are now proposing that the organization of the genome based on 10 nm fibres is sufficient to describe the organization of the DNA within the nuclear volume [3]. They favour a model in which active and silenced compartments within the genome arise through variations in the packaging density of 10 nm fibres. On the other hand a recent study presented new evidence for the occurrence and arrangement of 30 nm fibres. The authors used cryo-electron tomography to examine the ultrastructure of chromatin fibres in erythrocytes [13] revealing a 2-start left-handed helical arrangement of nucleosomes with ~6.5 nucleosomes per 11 nm consistent with a cross-linker model. The authors speculated that the special conditions in the erythrocyte nuclei favour the structure, but that in other nuclei these structures may be formed in facultative heterochromatin or as transient structures during the initiation of transcriptional repression. Thus while chromatin is generally considered to show a variety of higher order structures, their nature is still the subject of considerable disagreement and debate (see e.g., [14]).

Chromatin has traditionally been divided in two distinct classes: euchromatin and heterochromatin [15]. Originally referring to its appearance in microscope images, these terms are taken to designate states of compaction and transcriptional potential, although the higher-order structural differences are still debated. Heterochromatin is very condensed and consists mainly of repetitive sequences whereas euchromatin is gene rich and less compacted, with irregularly spaced nucleosome arrays [16,17]*.* In nuclei of mammals and some other eukaryotes these domains are organized in the nucleus with heterochromatin mainly localized towards the nuclear periphery and euchromatin in the interior of the nucleus [18,19,20]. 

**Figure 1:**
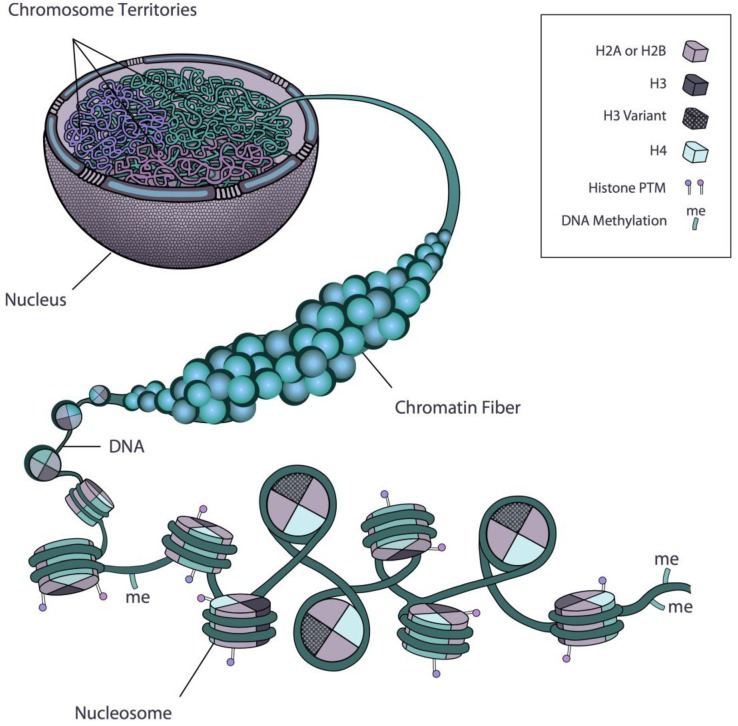
Organizational network of chromatin in the cell. Scheme depicting different aspects of chromatin regulation. PTM, post-translational modification. Chromosome territories within the nucleus, shown in different colours, are composed of chromatin fibres, which, in turn, contain packed nucleosomes.

The ability of a gene to be transcribed is thought to depend on its accessibility to the transcription machinery. In fact nucleosomes represent a barrier to many protein complexes that need to contact the DNA and regulate gene expression [21]. The mechanism by which the accessibility of genes to transcription can be regulated at the chromatin level involves two key aspects. One involves the disruption of interactions between nucleosomes or between the nucleosomes and the DNA, leaving the chromatin in a decondensed, ‘poised’ state favourable for transcription, while the other relies on the recruitment of non-histone chromatin binding proteins. In many cases these proteins have two or more domains, one of which recognizes the modified histone motif using a conserved protein domain, while the other domain exerts one of a variety of regulatory functions. Generally, these functions involve post-translational modifications of histone tails, incorporation of histone variants, or nucleosome sliding or remodelling by the activity of ATP-dependent remodelling complexes (Figure 1). Often both mechanisms are involved in specific gene activations, either sequentially or in parallel.

In this review we consider two main aspects of chromatin regulation at the nucleosome level (histone modifications and histone variants) as well as the spatial distribution of genes within the three-dimensional space of the nucleus as a means to regulate transcriptional activity.

## 2. Regulation of Gene Expression at the Level of the Nucleosome

### 2.1. DNA Methylation

DNA methylation at 5-methylcytosine (5-mC) is one of the best studied epigenetic marks implicated in regulation of gene expression. The function of DNA methylation includes roles in development and in genome defense against transposons. DNA methylation is confined to relatively specific regions of the genome. Highly methylated regions comprise: transposons, rDNA arrays and centromeric repeats. Heterochromatin knobs [22] and pericentromeric heterochromatin [23], which are rich in transposons and retrotransposons, are also strongly methylated.

In mammals, methylation is found almost exclusively in CG dinucleotides [24], with exception of embryonic stem cell and brain tissue where non-CG methylation seems to prevail [25,26] whereas in plants cytosines can be methylated in all possible sequence contexts including symmetric (CG and CHG, where H is A, C, or T) and asymmetric (CHH) [27,28,29]. Symmetric CG methylation is maintained by DNA METHYLTRANSFERASE 1 (MET1) [30], the plant homolog of mammalian DNA METHYLTRASFERASE 1 (DNMT1), which recognizes hemimethylated double stranded DNA (dsDNA) sustaining a pattern of methylation during DNA replication in a semi-conservative way. Methylation in non-symmetric CHH context is catalyzed by DOMAINS REARRANGED METHYLTRANSFERASES (DRM1 and DRM2) [31], the plant homolog of mammalian DNMT3a and DNMT3b. Methylation at CHH sites is targeted by 24-nt small interfering RNAs (siRNAs) as part of a pathway named RNA-directed DNA methylation (RdDM) [32,33,34,35,36]. Finally, methylation in the context of CHG is maintained by CHROMOMETHYLASE 3 (CMT3) [37], a plant-specific methyltransferase that recognizes dimethylated histone 3 tails at lysine 9 (H3K9m2). In this mechanism enzymes are recruited through direct recognition of the methylated histone H3K9me2 and do not require siRNA or other components of RdDM. Interestingly, a recent paper reported that a homolog of CMT3, CMT2, is implicated in maintenance methylation at CHH sites [38]. CMT2 is recruited through direct recognition of the methylated histone H3K9me2 (similarly to CMT3) and does not require siRNA guides or other components of RdDM, which was previously thought the only pathway leading to CHH methylation. Methyltransferases are fundamental for methylation to occur but normal DNA methylation also requires the chromatin remodeling ATPases DDM1 [39] and DRD1 [40], as well as methyl cytosine binding proteins VIM1, VIM2 and VIM3 [41].

Recent advances in high-throughput sequencing technologies combined with bisulfite treatment of Arabidopsis genomic DNA allow analyses and the generation of high resolution genome-wide methylation maps and are now revealing in unprecedented detail the patterns and dynamic changes of DNA methylation in plants. We will not discuss it further for the purpose of this article but there is currently a great body of literature on this subject [33,36,42,43,44].

### 2.2. Histone Modifications

Histone proteins contain flexible N-terminal tails that extend outward from the nucleosome core and that are subject to diverse post-translational modifications (PTMs). Although histone tails are the main targets for post-translational modifications, the histone core domains can also be modified (reviewed in [45]). For the purpose of transcription, histone modifications can be divided into two groups: activation-related marks and repressive marks. In general acetylation and phosphorylation have been correlated with activation while sumoylation, deimination, and proline isomerization have been associated with repressive states. Others like ubiquitination and methylation are correlated both with activation and repression [46,47,48]. These correlations led to the establishment of the histone code theory, which proposes that histone modifications act sequentially or in combination to affect gene expression [49].

In recent years the number of modifications that have been identified on histones has increased quite dramatically. Over eight different classes have been characterized to date and many sites have been described within each class. An additional degree of complexity is that multiple modifications can be added simultaneously in the same nucleosome or even on the same histone tail [50] creating an enormous number of regulatory possibilities*.* This suggests that the regulation is highly complex, rather than being a simple and predictable code in which each modification has a unique effect.

Histone modifications are caused by the direct action of histone-modifying enzymes. During the last few years, enzymes have been identified for almost all types of modification (reviewed in [51]). A number of proteins have been identified that are recruited to specific histone modifications. For instance, methylation is recognized by PHD (Plant Homeo Domain) fingers, whereas acetylation is recognized by bromodomains [51]. On the other hand some PTMs of histones have the potential to affect directly histone-DNA interactions playing a role in the accessibility the underlying DNA sequence and therefore in the regulation of gene expression.

Rapid progress in recent years has brought plenty of data about the diverse roles and mechanisms of histone modifications in regulating gene expression. For instance the arrival of genome sequencing brought the possibility to map genome modifications at high resolution and led ultimately to the concept of the “epigenome”. There are now several examples of epigenomic profiling in Arabidopsis [22,52,53,54,55,56,57] and a various microarray technologies together with high-throughput sequencing-based approached are available for genome-wide proﬁling of epigenetic modiﬁcations [58,59].

Not much is known, however, about the interplay between the mechanisms responsible for the addition and removal of these marks and how they are involved in orchestrating development. Further studies to reveal the molecular role of these factors will be necessary to understand the mechanisms underlying cell differentiation and development. 

#### 2.2.1. Histone Acetylation

The best characterized post-translational modification regulating chromatin structure is the acetylation of one or more of the lysine residues in the histone N-terminal tails. Its regulatory role was proposed about 50 years ago [60]. Histone acetylation is thought to facilitate transcriptional activation either by forming a binding site for bromodomain-containing proteins or by neutralizing the charges of the basic tails that interact with the acidic DNA [61]. A correlation between histone acetylation and transcriptional activity was shown for instance by immuno-labelling studies where antibodies specific to acetylated histones were used, revealing differential labelling in different chromosomal regions [62,63]. In animals, immuno-labelling of metaphase chromosomes with antibodies against acetylated H4 [64,65] and H3 [66] revealed that regions enriched in coding DNA were more strongly labelled, in comparison with unlabelled heterochromatin.

Of all modifications acetylation has the highest potential to induce chromatin unfolding. The neutralization of the positive charge of lysine at the histone tails has a strong effect in destabilizing inter-nucleosome contacts and therefore the chromatin structure itself. Experiments in which tail residues were chemically modified on recombinant histone core preparations showed that acetylation of histone H4 on lysine 16 (H4-K16Ac) has a negative effect on the formation a higher order chromatin structures *in vitro* [67]. 

The enzymes that acetylate histones, the histone acetyltransferases, are classified into three main families, GNAT, MYST and CBP/p300 [68]. In general these enzymes can modify many different lysine residues but some are specifically limited to certain residues (reviewed in [51]). Histone acetylation can be reversed by histone deacetylase enzymes, which remove acetyl groups from the acetylated histone tails. Their activity is correlated with chromatin condensation and gene repression [69]. The acetylation status of a given gene is therefore controlled by the balance between the activity of histone acetyltransferases (HATs) and histone deacetylases (HDACs).

In wheat (*Triticum aestivum)* inhibition of HDAC activity by trichostatin-A (TSA) was shown to affect gene positioning and the overall structure of interphase chromosomes [62]. In this species the interphase chromosomes are very well organised; the two chromosome arms of each chromosome occupy elongated territories spanning across the nucleus with the centromeric and telomeric regions lying at opposite sides of the nuclear membrane, defining a so-called Rabl configuration [70,71]. However in wheat seedlings germinated in the presence TSA, which induces histone hyperacetylation, the well-defined structure described above was disturbed with the chromosome arms becoming widely separated. Despite these changes, the Rabl disposition of interphase chromosomes was not altered by germination in TSA, suggesting strong interactions between the centromeric and telomeric regions with the nuclear membrane, which are not altered by acetylation levels.

#### 2.2.2. Histone Methylation

The modification of histones by methylation has been known for many years and there is currently a vast literature on the subject. Because of space constraints, we cannot discuss all aspects of all histone methylation. Nevertheless given its relevance in chromatin regulation we are still mentioning some general features. Histone methylation marks can be correlated both with repression and with transcriptional activation. In contrast to histone acetyltransferases, histone methyltransferases are highly specific and may be restricted to modification of one single lysine in a single histone [72]. For example, the lysine residues H3K4, H3K36 and H3K79 are methylation sites implicated in transcription while H3K9, H3K27 and H4K20 are methylation sites correlated with transcriptional repression. Additionally, lysine residues can be mono-, di- or trimethylated and each of these forms can have unique biological functions. For example, in the budding yeast, dimethylated H3K4 occurs at both active and inactive genes whereas trimethylated H3K4 is present exclusively in active genes [73]. Many proteins have been identified that are able to recognize specific histone modifications. The isolation of several proteins capable of recognizing H3K4me highlighted their role in tethering enzymatic activity onto chromatin. For instance the chromo-containing HP1 protein is recruited to H3K9me sites and brings with it deacetylase activity, which in turn prevents the accessibility of the underlying DNA sequence to the transcription machinery [74].

In plants the LIKE HETEROCHROMATIN PROTEIN 1 (LHP1), also known as TERMINAL FLOWER 2 and considered to accomplish the role of Polycombs (PcG) in plants, has the ability to bind to H3K27me3 [75,76]). LHP1 binding to H3K27me3 is required for its function [77], including the repression of several PcG protein targets such as *FLOWERING LOCUS C* (*FLC*) [78].

Histones can also be mono- or dimethylated on arginines [79]; however, much less is known about the effects of histone arginine methylation on chromatin regulation.

### 2.3. Histone Variants

Histone modifications and their implications for chromatin structure and regulatory processes that act on DNA, such as replication, repair and regulation of gene expression, have long been recognized. Similarly to histone modifications, the incorporation of histone variants into chromatin contributes to its regulatory repertoire. The expression of most of histones is tightly coupled to S phase of the cell cycle, when the newly synthesized DNA requires additional histone molecules [80]. These histones are generally designated “canonical” histones and in most metazoans are found clustered in repeat arrays [81]. In plants, core histones, although also present in several copies, tend to be dispersed rather than clustered. Histone variant genes are found dispersed in the genome and are expressed through all phases of the cell cycle in both plants and animals [82]. These variants possess particular characteristics that can dictate the switching on and off of the genes they are associated with, as well as other roles including DNA repair, meiotic recombination and chromosome segregation.

With the exception of histone H4, all of the core histones have a number of variants, which probably arose through gene duplication [82]. Here, we will focus on the histone variants with widespread occurrence and that have been so far better characterized—cenH3, H3.3 and H2A.Z. The evolutionary origin of these histones dates back to the earliest known diversifications of eukaryotes [81] and they can alter fundamental properties of the chromatin. However the molecular mechanisms and the specific integration pathways are not yet fully understood.

#### 2.3.1. CenH3

The cenH3 is an H3 variant that is specialized for packaging chromatin at eukaryotic centromeres and is essential for the assembly of kinetochores [83,84,85,86]. Centromeres are specialized chromatin structures within eukaryotic chromosomes that ensure their correct segregation at mitosis. Thus the presence of cenH3 is essential for cell division, acting as an epigenetic mark to specify the positions of centromeres and their perpetuation from one generation to the next. 

CenH3 variants have unusual features. Relative to the canonical H3, which is one of the most conserved proteins, cenH3 sequences are surprisingly divergent. Sequence analysis has revealed 50%–60% similarity with the canonical form at the C-terminal histone-fold domain (HFD) and little or no conservation at the N-terminal region [87]. In spite of these differences the function of cenH3 in organizing the kinetochores seems to be universal. Recent studies revealed that yeast cenH3 (Cse4) can substitute human centromeric H3 (CENPA) [88]. In this study, CENPA depleted cells induced by RNA interference were functionally complemented by expression of yeast Cse4. These results showed that there are conserved features in the structure of cenH3 nucleosomes that account for their universal role in forming the centromeres among different organisms. 

The dynamic behaviour of cenH3 was studied in Arabidopsis by developing transgenic lines in which this variant was tagged with GFP [89]. Using four-dimensional (4-D) live cell imaging, it was shown that the centromeres are constrained at the nuclear periphery during interphase but that global centromere position is not precisely transmitted from the mother cell to daughter cells. 

CenH3-containing nucleosomes have been traditionally considered as octamers; however recent studies suggest that they might be organized as tetramers. Evidence for that comes from MNase assays in Drosophila [90]. Unlike canonical nucleosomes that are wrapped around ~150 bp of DNA, cenH3 is wrapped around smaller sequences. Additionally, measurements by atomic force microscopy showed that CID (*Drosophila melanogaster* cenH3) nucleosomes have only half of the height of canonical nucleosomes. Together these observations support a model in which cenH3-containing nucleosomes consist of stable heterotypic tetramers (cenH3, H4, H2A and H2B)—the “hemisome” model [90]. However this composition is still a matter of debate. For instance, for the budding yeast cenH3 (Cse4), there are at the moment three models for the composition of cenH3-containing-nucleosome: the octasome, the hemisome and the hexasome models [91].

Another potentially important feature of cenH3 variants is that in common with archeal histones, they induce positive supercoils in DNA [92]. This positive coiling is proposed to resist the mitotic negative coiling of chromosomes during mitosis. This feature might be important in that it allows the centromeres to be accessible to kinetochores and kinetochore proteins during chromosome segregation [81].

#### 2.3.2. H3.3

Besides cenH3, other H3 variants have been reported. H3.3 differs from the canonical H3 only at four amino acid residues and is incorporated into chromatin in a replication independent fashion [80]. Three of these amino acid substitutions have been shown to be important for replication independent incorporation into chromatin. Mutation in any of these three residues was shown to be sufficient to confer partial replication independent activity to the canonical H3 [80]. 

Additionally, while the canonical H3 is incorporated in chromatin by the chromatin-assembly factor 1 (CAF1) during DNA replication and repair, H3.3 is assembled into chromatin by another complex—histone regulator A (HIRA)—independently of DNA synthesis [93]. In spite of the close similarity in amino acid sequence with the canonical form, H3.3s exhibit distinct posttranslational “signatures” that in turn, influence epigenetic states during cellular differentiation and development. This variant is enriched for the presence of marks, such as di- and tri-methylation of K4, acetylation at K9, K18 and K23, and methylation of K79, that reflect transcriptional competence [94]. H3.3 is therefore present at active loci as previously suggested by the observation that H3.3 was incorporated into active rDNA [80]. To examine H3.3, many groups have made use of tagged versions of the protein to perform chromatin immunoprecipitation (ChIP) and have shown that it is generally present at actively transcribed regions [95,96,97]. In addition, it has been shown that fluorescently labelled H3.3 is incorporated *in vivo* into a transgene array as the array undergoes activation [98].

Studies in *D. melanogaster* revealed that H3.3 is turned over more rapidly than H3 [99], which might contribute to keeping chromatin accessible in transcribed genes. It was also shown recently that nucleosomes containing tagged H3.3 in chicken cells are more sensitive to salt based disruption than those containing the canonical H3 [100]. A possible explanation for the reduced stability of H3.3 containing nucleosomes comes from the fact that three amino acid residues that differ from the canonical H3 do not make contact with H2A or H2B, leaving those nucleosomes containing them more susceptible to disruption by cellular processes such as transcription, chromatin remodelling and modification [81]. On that basis, H3.3-containing nucleosomes seem to be intrinsically less stable and this may reduce the energy needed to move or displace nucleosomes from promoter, enhancers and gene-coding regions.

#### 2.3.3. H2A.Z

H2A.Z is a highly conserved variant of the canonical histone H2A. Phylogenetic analysis indicates that H2A.Z diverged from the canonical form early on during the evolution of eukaryotes but since then it has been fairly well conserved. In fact, the H2A.Z variants of different organisms show a higher degree of sequence similarity (approximately 90%) than the H2A.Z and H2A within the same organism [101,102]. 

The H2A.Z variant has been widely studied and has been shown to be essential in both mammals and in Drosophila [103,104]. In *Saccharomyces cerevisiae* it is not essential for viability but has a function that cannot be substituted by the canonical H2A [105]. Unlike the single, non-essential H2A.Z gene from yeast, higher organisms have several H2A.Z genes. Vertebrates have two H2A.Z genes (H2A.Z1 and H2A.Z2) which encode proteins that differ by three residues [106]. In *Arabidopsis thaliana* there are three different H2A.Z genes (HTA8, HTA9 and HTA11), which are redundant to some extent and differ in cell-cycle regulation [107].

The H2A.Z variant is incorporated into nucleosomes by the action of a specific multi-subunit complex termed SWR1 in yeast and SRCAP in humans. SWR1 is an ATP-dependent remodelling complex that exchanges H2A-H2B dimers for H2A.Z-H2B in the nucleosomes [108,109,110,111]. Little is known about how H2A.Z is incorporated into specific chromatin regions, but it has been suggested that the bromodomain factor 1 (BDF1) binds to acetylated histone tails, targeting the SWR1-H2A.Z complex to chromatin containing acetylated H3 and H4 [112]. PIE1 is the homologue of Swr1 in Arabidopsis [113], and ARP6 and SEF are homologues of Arp6 and Swc6, two conserved subunits of the Swr1 complex in yeast, that, while not being essential, are required for the optimal function of the complex [114,115,116,117].

In *Tetrahymena thermophila* H2A.Z was found to be associated with the transcriptionally active macronucleus but absent in the transcriptionally inert micronucleus [118]. These observations suggested for the first time that this variant had a function related with transcription*.* The crystal structure of H2A.Z-containing nucleosomes suggested that its incorporation destabilises the interface between H2A.Z-H2B and the H3-H4 dimers [119]. Accordingly chicken nucleosomes reconstituted with H2A.Z were also less stable when assessed by sedimentation at different ionic concentrations [120]. A separate series of studies has concluded, in contrast, that the incorporation of H2A.Z into chromatin leads to more stable nucleosomes; on the other hand it impedes oligomerization of chromatin fibres suggesting that H2A.Z may create a transcriptionally poised higher order chromatin domain [121,122].

Studies of the properties of H2A.Z-containing nucleosomes have led to conflicting conclusions over the past few years. It appears that in spite of great sequence conservation, the function might have diverged considerably, with different functions described for different organisms [123,124]. In *Saccharomyces cerevisiae* H2A.Z has been implicated in diverse biological processes including the control of gene expression [125,126,127,128,129], cell cycle progression [130], chromosome segregation [131], DNA repair [132] and prevention of heterochromatin spreading to euchromatic regions [133]. In higher eukaryotes H2A.Z has roles in suppression of antisense RNAs [134], embryonic stem cell differentiation [135] and antagonizing DNA methylation in plants [136]. Its function, however, is not well understood and it has been associated with both euchromatin and heterochromatin.

H2A.Z can show contradictory functions even within the same organism. In differentiated mouse fibroblasts H2A.Z is distributed across the entire interphase nucleus but is excluded from transcriptionally silent and HP1 enriched pericentromeric regions [137]. However in trophoblast cells of the developing mouse embryo, H2A.Z appears to be concentrated at the heterochromatic pericentric regions and colocalizes with HP1 [138], suggesting that H2A.Z role switches at different stages of development and differentiation. 

Recent studies have suggested one reason why H2A.Z function shows such contrasting effects. Genome-wide ChIP sequencing or ChIP-microarray experiments showed that H2A.Z preferentially localizes in the 5' regions of genes within euchromatic regions [128,139,140,141,142]. It was suggested that the deposition of H2A.Z at the 5' end of genes may act to set up an architecture that is compatible with gene regulation at promoters [143]. In mammalian cells, mapping of DNA hypersensitive sites, based on the activity of micrococcal nuclease digestion, has been used to define open chromatin regions of the genome, which often correspond to enhancers and promoters [144]. Overlaying these sites with ChIP-chip data from T cells revealed an enrichment of H2A.Z at DNAse hypersensitive sites [142]. Therefore the presence of H2A.Z at promoter regions might act so as to maintain an open chromatin structure that in turn allows the binding and action of either transcriptional activators or repressors. This feature of H2A.Z together with differential effects of PTMs or the association with other variants like H3.3 [100] may be a possible explanation for the dual behaviour observed of this variant.

Finally, research in Arabidopsis presented a new concept where H2A.Z-containing nucleosomes provide thermosensory information for coordinating temperature responses in plants [145]. H2A.Z nucleosome occupancy decreases with increasing temperature, independently of local transcription levels. The authors thus propose a model where the tight H2A.Z-nucleosomes are loosened up by higher temperatures. This study suggest that temperature perception may take place at the chromatin level, with H2A.Z-containing nucleosomes monitoring the shifts in ambient temperature and executing the corresponding alterations in global gene expression. A recent study, also in Arabidopsis, revealed an interesting anti-correlation between H2A.Z and DNA methylation. Moreover the authors suggest that H2A.Z deposition in gene bodies generates variability in gene expression, and that a function of gene body methylation is to exclude H2A.Z from constitutively expressed genes [146].

### 2.4. ATP-Dependent Remodelling Complexes

As well as modification either covalently by histone post-translational modifications or by incorporation of histone variants, nucleosomes can also be mobilized to different positions along the DNA, or evicted by ATP-dependent remodelling enzymes. ATP-dependent remodelling factors play an essential role in gene expression by regulating the access of the transcription machinery to DNA sequences [147]. Generally they are composed of several subunits (up to 15 subunits) one of them being an ATPase belonging to the SNF2 superfamily of DNA helicase/ATPase. The ATPase-containing subunit is embedded in a multiprotein complex that mediates different nucleosome remodelling activities [148]. A number of ATP-dependent chromatin remodelling enzymes have been identified. All eukaryotes contain at least 5 types of ATP-dependent remodellers: SWI/SNF, ISWI, NURD/Mi-2/CHD, INO80 and SWR1 [147].

Current data from studies in animals suggest that the biochemical mechanism by which each type of remodeller acts is distinct and may account for their unique biological functions. For example SWI/SNF complexes are able to induce ATP-dependent disruption of nucleosome structure, facilitating the binding of transcription factors to their target sites on nucleosomal templates [149]. In contrast, the ISWI members relocate nucleosomes by sliding the histone octamers along the DNA template [148]. The SWI/SNF subfamily functions mainly in transcriptional activation or repression, while the ISWI subfamily has additional roles in maintaining stable higher-order chromatin structure and chromatin assembly. Despite the different outcomes, common mechanisms seem to underlie their action. These mechanisms include both the unwrapping of DNA segments from the nucleosome core and the translocation of DNA loops through the nucleosomes [150,151]. 

An obvious question is how these ATP remodellers are targeted to the appropriate genomic regions. Different mechanisms have been proposed; the best studied is based on targeting via DNA-sequence-specific factors [152]. For instance the ISW2 is targeted to the promoters of early meiotic genes by the transcriptional repressor UME6 [153]. The consequence is the formation of an inaccessible chromatin structure that is not compatible with gene expression. The interaction between transcription factors and ATP-dependent remodelling enzymes can also be a mechanism for targeting to specific sites, as described for the interaction between SWI/SNF proteins and the basic-Helix-Loop-Helix (bHLH) proneural transcription factors in neurogenesis [154]. Other modes targeting ATP-dependent nucleosome remodelling factors that have been proposed include the interaction with methylated DNA and intergenic RNA transcription [155,156].

Another way by which ATP remodelers regulate chromatin structure and gene regulation is via incorporation of histone variants. One example, mentioned previously, is the ATP-dependent remodelling enzyme responsible exchanging the canonical histone H2A with the variant H2A.Z. The SWR1 complex is named after its ATPase sub-unit Swr1 (for Swi2/Snf2 related). This complex seems to be targeted to specific sites by a mechanism that involves the recognition of histone acetylation. This conclusion came from the identification of Bdf1 (*Bromodomain factor 1*) as a member of the SWR1 complex [108,157]. An interesting study revealed that the absence of a functional Bdf1 protein has a similar phenotype as absence of SWR1 or H2A.Z [112]. However, it is not yet clear whether Bdf1 is able to recognise specific acetylation motifs, or what sets the signal in the first place.

## 3. Regulation of Gene Expression by Higher-Order Chromatin Organization

Although the nucleosomes represent the basic unit of chromatin compaction, they constitute only the first level of chromatin condensation. The amount of condensation needed for a typical genome to be fitted into an interphase nucleus or set of metaphase chromosomes, indicates that there are additional “higher order” levels of chromatin organization. In the context of chromatin, higher-order structure may be defined as any reproducible conformation of nucleosomes in 3D space*.* The most evident form of higher-order chromatin structure is the mitotic/meiotic chromosome in which the DNA is highly compacted. Studies on 3D spatial locations and transcriptional competence of genes in respect to their chromosome territories have provided some important insights on the importance of this level of chromatin organization on regulation of gene expression.

### 3.1. Chromosome Territories

While chromatin organization in interphase nuclei has been a topic of speculation for many years, a lot of progress has been made recently. The idea that chromosomes occupy distinct regions in the interphase nucleus when they are decondensed during interphase was first proposed by Carl Rabl in 1885 [71] and later developed by Theodor Boveri, who applied the term chromosome territory (CT) for the first time [158]. Years later advances in *in situ* hybridization techniques using ‘chromosome paints’—mixtures of *in situ* probes made from the non-repetitive regions of entire chromosomes—finally allowed the direct observation of distinct non-overlapping chromosome territories [159,160]. 

**Figure 2:**
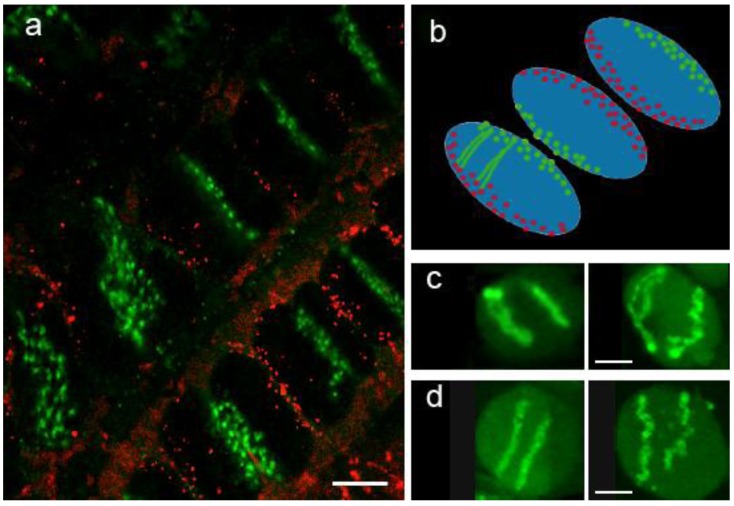
Rabl chromosome organization in wheat root tissue. (**a**) Centromeres (green) and telomeres (red) are labelled by fluorescence *in situ* hybridization (FISH) and are located at opposite sides of the nuclei; (**b**) Diagrammatic interpretation of the organization in (**a**); (**c**) Introgressed pair of rye chromosomes in wheat labelled by genomic *in situ* hybridization (GISH) with total rye genomic probe confirms a Rabl organization of individual CTs. (**d**) Single rye arm translocation into wheat localized by GISH. In (**c**) and (**d**) the RH panel in each case shows a nucleus from a seedling treated with 5-AC, whereas the LH panel shows a control untreated seedling. The 5-AC treatment has disrupted the CT organization, but the CTs remain in a Rabl configuration. Bar = 10 µm.

The high content of dispersed repeats in plants species with large genomes for many years prevented the development of suitable chromosome paints for plant species. As an alternative, interspecific hybrid lines, carrying one or more pairs of alien chromosomes were used to provide the first images of plant chromosome territories ([70,161] see Figure 2c,d). The complete genome of the alien species was used to make a probe that specifically revealed the alien chromosomes. These probes are generally taken to target species-specific repetitive sequences. One objection to this approach was that the alien chromosomes may not be behaving in the same way as the native chromosomes. Chromosome territories in plants were ﬁrst visualized using chromosome paints in Arabidopsis using chromosome-speciﬁc mixed bacterial artiﬁcial chromosome (BAC) FISH probes [162].

While not much is known about what controls the organization of CT in interphase it is believed that several factors might be involved, including content and distribution of heterochomatin and repetitive regions, association with the nuclear envelope among others [163]. In many plant species containing large genomes (e.g., wheat, oat, barley, rye), the interphase CTs often adopt a Rabl conformation. In the Rabl configuration the chromosomes fold back at their centromere, so that the telomeres at both ends of the chromosomes are located together (see Figure 2). This conﬁguration is thought to be a default organization where chromosomes maintain the preceding anaphase orientation. However in other species such as maize (*Zea mays)*, sorghum (*Sorghum bicolor*) and Arabidopsis this arrangement is not observed, and chromosomes seem to lose their polarized anaphase configuration after entering interphase. In Arabidopsis, the telomeres have a strong tendency to be associated with the nucleolar periphery, and this association has been suggested to be an initial stage in meiotic homologue pairing [164].

In rice (*Oryza sativa*) the Rabl congfiguration is only observed in certain tissues [165] but treatment with 5-azacytidine (5-AC), a DNA hypomethylating drug, was shown to induce a Rabl conﬁguration in those tissues in which the Rabl configuration is not normally present [166]. This suggests that changing the methylation state of the chromosome can alter the overall CT configuration. This was confirmed by the changes observed in the chromosome territories seen in wheat interspecific hybrids [62]. In this case, the overall Rabl configuration of the chromosomes is maintained after treatment with 5-AC, but the CT becomes irregular, and appears fragmented, presumably because of regions of extensive decondensation (see Figure 2c,d).

The discovery of chromosome territories led to intriguing questions, for instance: is the position within the nucleus, and the position relative to each other, an inherent property of chromosomes, cell types and tissues? Is such chromosome positioning a cause or consequence of their gene expression state? The emerging view is that the location of a gene within a chromosome territory seems to influence its access to the machinery responsible for specific nuclear functions, such as transcription. One typical example of relocation within a chromosome territory is the ANT2 gene located on the X chromosome. When present on its inactive homolog, this gene is found in the interior of the chromosome territory; however, its position changes on the active homolog where it can be found at the periphery of the territory [167]. This observation of an inactive gene being located in the middle of a chromosome territory and its relocation towards the periphery by a “looping out” mechanism has been shown several times [168,169]. Nevertheless counter observations have also been reported. For instance, in the case of wheat roots transcription sites revealed by BrU incorporation were not preferentially localized with respect to the chromosome territorial boundaries but uniformly distributed throughout the nucleoplasm [70].

**Figure 3:**
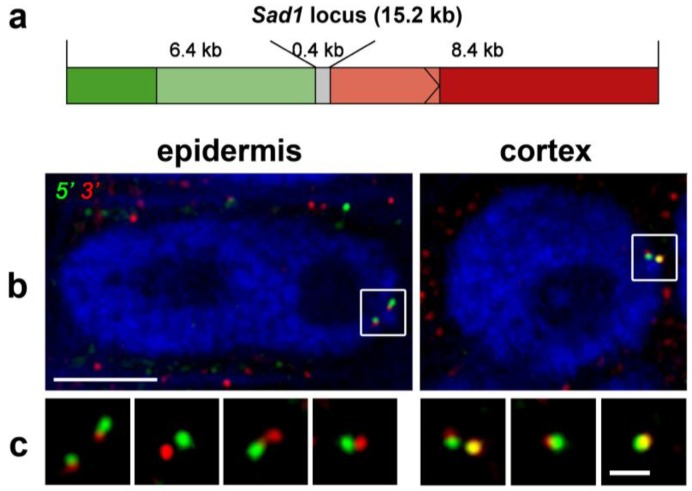
Decondensation of the Sad1 gene locus in oat root visualized using different coloured probes for the 5' and 3' ends of the gene. (**a**) Diagram of the labelling scheme used; the 5' portion is labelled in green, the 3' portion in red; (**b**) Examples of G2 nuclei from tissue labelled with the two probes. In the epidermal cells, where Sad1 is actively transcribed, the red and green ends of the gene can be seen to be separated, whereas they overlap to a much greater extent in the cortex cells, where Sad1 is not transcribed. Bar = 5 m; (**c**) A gallery of Sad1 double labelled sites from different nuclei. Bar = 1 µm.

Studies on clusters of functionally related genes have also revealed transcription-related chromatin decondensation events in mammals. Interestingly, examples of secondary metabolite gene clusters have now also been discovered in plants. For instance in oat (*Avena strigosa*) the activation of the avenacin gene cluster is accompanied by chromatin decondensation [170]. The authors focused on *Sad1* and *Sad2* genes and used mRNA and DNA *in situ* hybridization techniques to show that the two genes moved apart from approximately 0.45 to 0.90 µm upon activation in a cell type-specific manner. They also showed that individual genes elongated on activation to the order of about 20-fold compaction compared to naked B-DNA. This is more compact than the 10 nm fibre, but less than the proposed 30 nm fibre (see Figure 3).

Movements at larger scale have also been reported. Loci undergoing changes in transcriptional status as part of a developmental program can relocate to specific regions of the nucleus. An example of this scenario was provided by the nuclear reorganization that accompanied the differentiation of mouse lymphocytes [171,172], where lymphoid genes targeted for silencing are relocated to pericentromeric heterochromatin clusters. However it appears that, at least in this example, transcriptional repression precedes the relocation, suggesting that relocation is not vital for transcriptional repression of the gene. In contrast, genes marked for active transcription were shown to move away from centromeric chromatin [173] or the nuclear periphery [174]. Although there is strong evidence regarding the correlation between intra-nuclear or intra-territory location with transcription, the chromatin conformational changes that follow the switch in transcriptional status are still far from being understood.

Although it seems in principle unlikely that the large transcription machinery complexes could access DNA and operate in compact chromatin, making the concept of the chromatin looping to facilitate transcription attractive, the fact is that highly compact chromatin can still be accessible in some extent. A recent study designed to examine the compaction state of active and inactive chromatin in interphase nuclei has indicated quite small transcription-related changes in higher-order structure, implying that transcription can proceed in quite compact chromatin [175]. In Arabidopsis, 3D fluorescence *in situ* hybridisation in well-preserved root tissue was used to examine chromatin reorganization around the *GLABRA2* locus [176]. The authors were able to see that in cells where the locus is inactive the labelling intensity was much lower, suggesting that accessibility and chromatin compaction might underlie the transcriptional state of a given locus. 

These observations raise the question of how transcription can proceed in a compact chromatin structure. In terms of accessibility, several studies have shown that rather large molecules can penetrate compact chromatin structures [177,178,179]. This suggests a new view of chromatin behaviour in which it is regarded as a highly dynamic structure where binding sites are continuously being scanned by chromatin proteins in a random way. Novel approaches to study these and other dynamics aspects of chromatin such as live cell imaging and photobleaching are discussed below.

Another possibility is the occurrence of discrete sites for transcription in the nucleus, which have been termed transcription factories [180]. According to this model DNA and RNA polymerases are immobilised by attachment to an underlying nuclear substructure or matrix [180]. These polymerases are suggested to be concentrated in discrete factories, where they work together on many different templates (reviewed in [181]). This view arose from visualization of nascent transcripts by BrU incorporation as a series of discrete foci, many fewer in number than the presumed number of transcribed genes. It has some support from recent data produced by technical advances in fluorescence *in situ* hybridization (FISH) and immunofluorescence-FISH, and in molecular approaches such as chromosome conformation capture (3C) and related methods [182,183] that have the power to scan the genome for sequences or regions that are positioned close together in nuclei. Even though this is an attractive model it still lacks direct evidence, however, and the existence of transcription factories remains a matter of debate (reviewed in [184]). 

### 3.2. Chromatin Conformation Capture (3C)

The development of new molecular, genomic and computational approaches based on chromosome conformation capture technology (such as 3C, 4C, 5C and Hi-C) are currently helping us to study the spatial organization of genomes at an extraordinary level resolution. In 3C-based methods cells are initially cross-linked with formaldehyde to preserve chromatin structure and subsequently digested with restriction enzymes or sonication. Cross-linked fragments are then ligated resulting in segments that contain sequences that were in close spatial proximity. The DNA is then finally purified and analysed.

The different 3C-based methods differ essentially in the way the DNA segments are detected and quantified. In conventional 3C techniques, single ligation products are detected by PCR one at a time using primers from specific sequences. 4C, chromosome conformation capture-on-chip, combines 3C technology with microarrays to analyse the contacts of a specific genomic site with all of the genomic fragments present on the array generating therefore a genome-wide interaction profiles for single loci [185,186]. In “3C-Carbon Copy” or 5C a multiplexed ligation-mediated amplification (LMA) is used to copy and amplify parts of the 3C template, the readout of these occurs either on a microarray or by high-throughput sequencing [187]. The Hi-C method maps interactions in complete genomes in an unbiased fashion [188]. It combines the use of restriction enzymes that leave 5' overhangs with a step in which the DNA ends are filled in with biotinylated nucleotides. Biotin pull-down and sequencing enables the detection of ligated segments providing a genome-wide interaction map. We will not discuss these techniques further in this review. However 3C-based methods have been extensively reviewed and discussed in recent publications [189,190,191].

## 4. Live-Cell Imaging Approaches to Studying Chromatin Dynamics

For many years the majority of studies that examined the mechanisms by which chromatin regulates transcriptional activity focused on measuring steady-state levels. A typical biochemical approach is chromatin immunoprecipitation, in which chromatin fragments still associated with histones or other chromatin proteins (after formaldehyde cross-linking) are precipitated using a specific antibody, and the DNA or proteins associated with the target are then analysed. The trouble with these types of approach is that often they do not take into account the dynamic properties of chromatin, leading to rigid assumptions about stable interactions between chromatin and chromatin proteins *in vivo*. Recently, a combination of molecular biology and live cell microscopy techniques has allowed observation of the dynamic behaviour of chromatin *in vivo* within the natural environment of the nucleus. 

### 4.1. Measuring Histone Mobility—FRAP

The first experimental evidence to indicate that the protein composition of chromatin was not static came from radioactive isotope labelling studies in the early 1980s [192,193,194]. These experiments clearly showed histones being exchanged with pre-existing histones in the chromatin. However, this method did not allow a precise measure of the exchange rates and could not demonstrate exchange *in vivo*.

New techniques such as fluorescence recovery after photobleaching (FRAP) confirmed that most of the functionally important characteristics of chromatin show a dynamic behaviour. With the development of confocal microscopes and their laser illumination systems, FRAP became a very accessible technique for analysing the kinetics of molecules in living cells (FRAP is reviewed in [195,196]). FRAP experiments require that the protein of interest is tagged with a fluorescent label (usually the green fluorescent protein, GFP, or a related tag) and expressed in a living cell. A typical FRAP experiment consists of three phases. First, the initial fluorescence intensity is measured, after which a high power laser pulse is used to photobleach the fluorophore in a small region of interest (ROI), and finally the recovery of the fluorescence intensity in that region is monitored in a time-course series. The exchange of non-bleached proteins for the bleached molecules in the bleached region produces a recovery of fluorescence intensity. In a situation in which all the molecules are immobile, the bleached area remains bleached and unbleached area unaltered, and no recovery is observed. On the other hand, if all the molecules are mobile and free to diffuse, the fluorescence in the bleached area recovers quickly to close to the original intensity levels. In a third situation, when both immobile and mobile fractions are present, the recovery curve reaches a plateau below the level before bleaching, depending on the relative abundance of the two fractions. The rate at which fluorescence is regained in the photobleached region is determined by the rate at which fluorescent molecules can move from the surrounding unbleached region into the bleached region, which is often determined in turn by the rate at which the bleached molecules dissociate from their binding sites. By analysing this rate, a diffusion constant can be determined.

The first results of FRAP experiments on histone H1-GFP fusions appeared in 2000 [197,198]. Both studies showed that instead of being permanently fixed in chromatin, the majority of H1 proteins were being exchanged, with a residence time of only a few minutes. In another study a number of nuclear proteins fused to GFP confirmed the earlier results on H1 and additionally demonstrated that a wide class of other nuclear proteins have also a high turnover rate [199]. These results indicated that chromatin-binding proteins find their binding sites by 3D scanning of the nuclear space and that this property might be crucial for generating high plasticity in genome expression.

Apart from histone H1 the mobility of many other histones has also been assessed by FRAP (reviewed in [200]). Compared to the linker histone H1, core histones have much slower exchange rates. Generally, core histones have a small pool of free proteins and another pool that is tightly bound to the DNA, with residence times of several minutes. H3 and H4 assemble to DNA during replication and only a minor population is exchanged independently of transcription and replication [201]. H2B and H2A, however, exhibit considerably higher exchange rates. In HeLa cell lines H2B seems to have different populations of molecules that exchange at different rates. There is a small population that exchanges rapidly, another population (~40%) that exchanges slowly (*t*_1/2_≅ 130 min) independent of ongoing DNA replication and transcription, and another (>50%) that remains bound stably (*t*_1/2_ > 8.5 h) [201]. 

The mechanism that accounts for the differences in kinetics between different histones and between the different pools within the same type of histone is related to differences in biochemical modifications. For instance it is known that acetylation of histone tails alters the stability of nucleosomes and also has been shown to alter the dynamics of H2B in living cells [202]. Acetylation of histone tails also appears to facilitate chaperone-mediated histone exchange; *in vitro* studies showed that the transfer of H2A–H2B from nucleosomes to histone chaperone Nap1 (nucleosomes assembly protein 1) is facilitated by p300-mediated acetylation [203]. Although we are only now beginning to understand the kinetics of different histone variants and FRAP analysis has yet to be carried out for the majority of the variants, it is very likely that these histones have different mobilities compared with the canonical forms and that their incorporation has the potential to affect of histone and nucleosome stability as already suggested by biochemical approaches.

Recently the development of photoswitchable or photoactivatable tags has made them an optimal tool for studying the spatial and temporal dynamics of proteins *in vivo* [204]. As the activation/conversion process is quicker than bleaching, it is more suitable than FRAP for measuring fast diffusion kinetics. Furthermore, it is easier to see a high signal against a low background than a low signal against a high background, as with FRAP. Another advantage of photoactivation/conversion is that much lower laser intensity is required for activation or conversion than with bleaching, thus substantially reducing photodamage to cells. Additionally, as the fluorescence of these proteins is seen only after photoactivation, newly synthesized non-photoactivated pools are not observed and do not complicate experimental results. This signal independence from new protein synthesis also allows the study of protein degradation of tagged molecules by “optical pulse labelling” and monitoring of the fluorescence over time.

An alternative method for measuring histone turnover showing great promise has recently been developed [205]. The method involves metabolic labelling followed by capture of newly synthesized histones, and has been termed Covalent Attachment of Tagged Histones to Capture and Identify Turnover (CATCH-IT). In this approach, the authors treated *Drosophila melanogaster* S2 cells with the methionine (Met) surrogate azidohomoalanine (Aha). The surrogate amino acid is incorporated into histones, and Aha coupled with biotin. Streptavidin beads are then used to affinity purify nucleosomes containing newly synthesized histones. The nucleosomal DNA is finally hybridized to a high-density tiling microarray to create a map of nucleosomes that have turned over during the pulse. The great advantage of this strategy is the possibility of assaying native histones, overcoming limitations related to requirements for transgenics and tags. The method should be applicable across species; however; there are only a few reports available so far.

### 4.2. Single Particle Tracking to Study Gene Positioning—Lac Operon System

While it is clear that spatial organization of chromatin in the nucleus is highly correlated with gene expression, the majority of the techniques used to study chromatin structure are unable to reveal a real dynamic view, providing instead a static analysis under a particular condition. The observation of chromatin motion requires the labelling of specific chromatin loci with techniques that are compatible with live cell imaging.

Recently, taking advantage of the exploitation of bacterial repressor/operator interactions in conjugation with GFP technology, significant breakthroughs in this field have been possible. Two systems are currently in use: one is based on the *lac* operator/repressor (*lac*O/LacI) [206,207,208], while the second one uses tet operator/repressor (*tet*O/TetR) [209,210]. Both systems rely on the genomic integration of tandem arrays of the relevant binding sequence in eukaryotic cells that express the bacterial repressor protein fused with GFP, which enables the tracking of specific chromosomal sites by real time fluorescence microscopy [211,212]. The locus is detected through the binding of the repressor protein, which is fused to GFP and has an added nuclear localization signal. The tagged sequence is seen as a bright dot in the nucleus, which preferably has a low general background fluorescent signal from the unbound GFP-LacI repressor (careful selection of the optimal expression level is often necessary to achieve a low background fluorescence). The movement of this bright dot can be followed and the measured trajectory can be analysed to obtain information about the mechanisms by which the tagged sequence is moving.

The initial surprise from this approach was the dynamic behaviour that chromatin exhibits in both yeast and *Drosophila* nuclei [213,214]. These studies showed for the first time that the GFP tagged chromatin loci undergo constrained Brownian motion within a limited sub-region of the nucleus during interphase. The amplitude of these movements seemed to be dependent on position within the nucleus; the chromatin associated with nucleolus or localized at the nuclear periphery was shown to be more restricted in its movements than other more nucleoplasmic genomic regions [215]. In another study, it was shown that sites with higher levels of transcription explored larger regions [216]. Studies in CHO cells showed that a specific DNA region changed its position from the periphery to the centre of the nucleus upon transcriptional induction by the transcriptional activator VP16 [217]. These observations raised several interesting questions: what is the significance of these movements? Is this mobility a result of an active energy-dependent process?

Experiments in yeast showed that early and late origins of replication were more mobile in G1 than in S phase, and that the movement in G1 was highly sensitive to ATP depletion and changes in metabolic status [218]. This correlation makes it unlikely that the movement results from a simple diffusion mechanism. Therefore it was proposed that the movement reflects the action of large ATP-dependent enzymes involved in transcription or chromatin remodelling. This hypothesis is consistent with the reduced mobility detected in stationary phase cells where transcriptional activity drops substantially [18].

**Figure 4:**
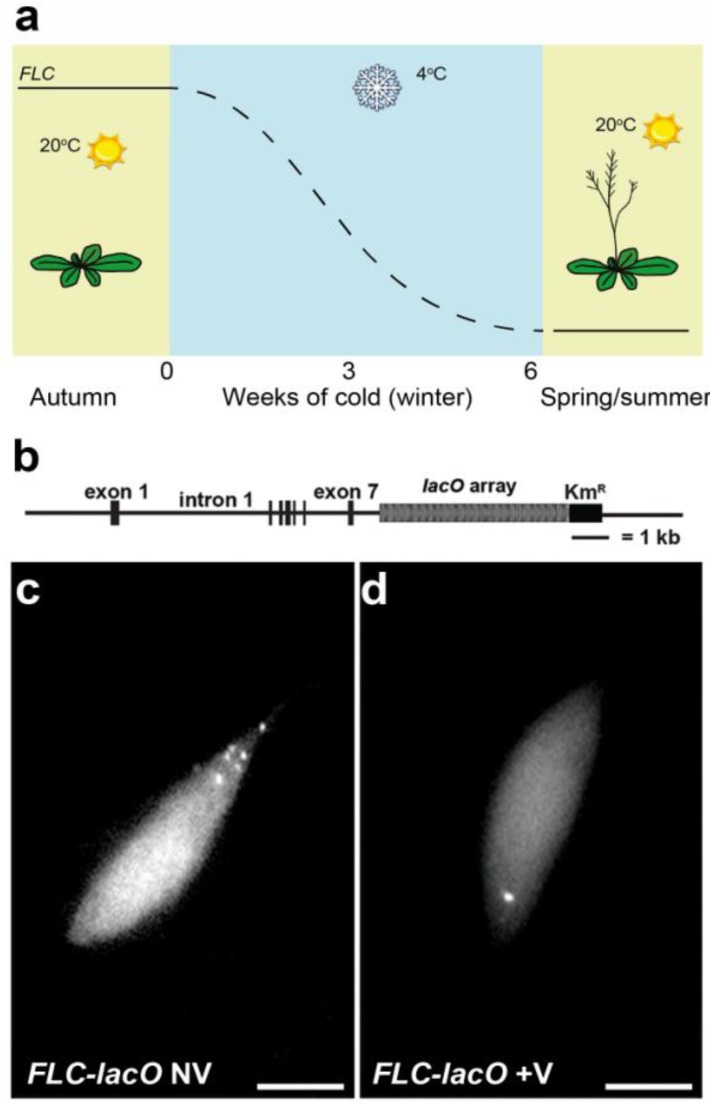
Vernalization in Arabidopsis involves Polycomb-mediated epigenetic silencing of *FLOWERING LOCUS C (FLC)* and physical clustering of *FLC* alleles. (**a**) Schematic representation of changes in FLC expression during vernalization; (**b**) *FLC* nuclear position was monitored in root nuclei using a *FLC-lacO* transgene. The *lacO* array was inserted downstream of the polyadenylation site of *FLC*; (**c**–**d**) Representative fluorescence images of Arabidopsis root cells in plants grown in warmth conditions (non-vernalized, NV) (**c**) and plants grown in cold for 2 weeks (vernalized, +V) (**d**). (scale bars: 5 μm).

In plants a few studies have used this approach. The first use [219] showed that chromatin in endoreplicated pavement cells had a greater range of movement than diploid guard cells in Arabidopsis. Several groups [220,221,222]) have developed sets of Arabidopsis lines labelled at various (random) chromosomal positions. In Arabidopsis the *lac* operator system has been used to analyse the repositioning of alleles of a Polycomb target gene, *FLOWERING LOCUS C (FLC)*, during an environmentally-triggered silencing process involved in the control of flowering time—vernalization [223]. This study revealed that alleles of the *FLC* locus physically associate and reposition in the nucleus during in the silencing process (see Figure 4), and the biological significance of this clustering was revealed by its dependence on trans factors shown to be required for the Polycomb silencing mechanism.

One drawback of this type of approach is the high sensitivity to photodamage and bleaching. It was shown for both yeast and mammalian cells that the movement of the tagged sequence is affected by long time-course imaging [224,225]. Thus, other types of microscopes such as two-photon microscopes (reviewed in [226]) have been suggested as possible alternatives for imaging with improved photostability of the sample [227].

## 5. Conclusions

In summary, as the field of chromatin dynamics has developed, it has become increasingly clear that a large number of underlying physical and molecular phenomena is involved, and that to fully understand them, information at many different levels is required. The study of chromatin dynamics and its effect on gene expression has opened new and exciting perspectives and one important goal of current research is to be able to relate these different levels of regulation. Advances and new strategies that allow “super” resolution in light microscopy will soon be in common use and may open the way to this type of correlative work.
